# Effect of Hot Rolling on the Microstructure and Mechanical Performance of a Mg-5Sn Alloy

**DOI:** 10.3390/ma15175973

**Published:** 2022-08-29

**Authors:** Xiaoru Zhuo, Cong Shao, Peng Zhang, Zhichao Hu, Huan Liu

**Affiliations:** 1 Nanjing Institute of Agricultural Mechanization, Ministry of Agriculture and Rural Affairs, Nanjing 210014, China; 2 College of Mechanics and Materials, Hohai University, Nanjing 211100, China; 3 College of Engineering, Nanjing Agricultural University, Nanjing 210031, China; 4 Jiangsu Key Laboratory for Light Metal Alloys, Nanjing Yunhai Special Metals Co., Ltd., Nanjing 211200, China

**Keywords:** Mg-Sn alloy, rolling, strength, ductility, strengthening mechanism

## Abstract

A Mg-5Sn alloy was hot rolled at 380 °C with three different rolling reductions (30%, 50%, and 70%), and its effect on the microstructure and mechanical performance was examined. Grain size decreases, whereas the area fraction of Mg_2_Sn particles and dislocation density increase with the increase in rolling reduction. Therefore, the yield strength (YS) and ultimate tensile strength (UTS) exhibit an ascending trend, whereas the elongation (EL) shows a descending trend with increasing rolling reduction. The alloy hot rolled for 70% possesses a high strength of 310 MPa and an EL of 8.4%. The strength enhancement is mainly ascribed to precipitation strengthening, grain refinement strengthening, and dislocation strengthening.

## 1. Introduction

Mg alloys are the lightest metallic structural materials with superior properties, such as excellent castability, good damping capacity, and high specific strength, making them ideal light-weight materials for applications in many industrial fields including automobiles and rail traffic [1,2,3,4,5]. It has been reported that every weight reduction of 100 Kg of a vehicle reduces the fuel consumption by 0.3–0.4 L/100 km [6]. Replacing the steel and Al alloys in vehicles and trains by Mg alloys can significantly reduce the consumption of energy and the emission of CO_2_, contributing to the alleviation of the energy crisis and global warming. However, the strength of Mg alloys is commonly lower than that required for application as structural materials. The addition of rare earth (RE) elements enhances the mechanical performance of Mg alloys, but substantially boosts the costs, restricting their application. Thus, it is very important to develop high-performance RE-free Mg alloys.

Mg-Sn alloys are deemed promising RE-free Mg alloys because they are similar to RE-containing Mg alloys in two aspects [7]. Firstly, the solubility of Sn in Mg is very high at high temperatures (3.35 at.% at 561 °C), and it reduces to a negligible value at room temperature, making Mg-Sn alloys age-hardenable [8,9,10,11,12]. Secondly, the intermetallic Mg_2_Sn phase in the Mg-Sn alloys possesses a very high melting temperature of 770 °C [8,9,13]. However, the mechanical performance of the as-cast Mg-Sn alloys is inferior. Thermomechanical processing has been employed to improve the mechanical performance of Mg-Sn alloys [8,14,15,16,17]. Hot extruded Mg-Sn alloys exhibit superior strength compared to their as-cast counterparts [14,15,18]. The strength enhancement is mainly attributed to grain refinement strengthening and precipitation strengthening. As Sn content increases from 1% to 7% (in wt.% unless otherwise noted), the YS and UTS of hot extruded Mg-Sn alloys increase, while EL decreases [18]. Similar results were reported by Cheng et al., in their study on the mechanical properties of hot extruded Mg-(6, 8, 10)Sn alloys [15]. Zhao et al., reported that YS, UTS, and EL increase as the Sn addition increases from 1.3% to 4.7% [19].

Note that previous studies on the hot working of Mg-Sn alloys are mainly focused on hot extrusion and rare attention has been paid to hot rolling. Due to the hexagonal close-packed (HCP) structure, the plasticity of the as-cast Mg alloys is poor [20]. When hot extruded, the Mg-Sn alloys are under a triaxial compressive stress state, which is conducive to improving workability [21]. In contrast, the Mg-Sn alloys are mainly under uniaxial compressive stress state during hot rolling and the workability is relatively poor. Therefore, the hot rolling of Mg-Sn alloys has rarely been reported. Hot rolling is a very efficient and cost-effective technique to fabricate Mg alloy plates with good performance [22,23]. It has been reported that hot rolling can effectively refine the microstructure and thus improve the mechanical performance of Mg alloys [22,24,25,26,27,28,29]. In this work, the effect of hot rolling on the microstructure and mechanical performance of a Mg-5Sn alloy was investigated. The results of this study may provide useful guidance for developing high-performance Mg alloys.

## 2. Materials and Methods

The raw materials for the fabrication of Mg-5Sn alloy are pure Mg (99.9%) and pure Sn (99.99%) ingots. They were melted in a zinc oxide-coated graphite crucible using an electric resistance furnace. Subsequently, the melt was cast in a zinc oxide-coated steel mold (with a dimension of 200 × 50 × 50 mm^3^) which was pre-heated to 300 °C. The melting and casting processes were conducted under the protective atmosphere of CO_2_ (99% in volume) and SF_6_ (1% in volume). Then, the as-cast alloy was machined into small pieces with a dimension of 19.5 × 42 × 3.5 mm^3^ through wire electrical discharge machining. Some of the small samples were solid-solution treated at 480 °C for 12 h, which were then hot rolled at 380 °C for three different reductions of 30%, 50%, and 70%, respectively. The samples hot rolled for 30%, 50%, and 70% were denoted as the R30 alloy, R50 alloy, and R70 alloy, respectively. The rolling reduction per pass is 10%.

**Figure 1:**
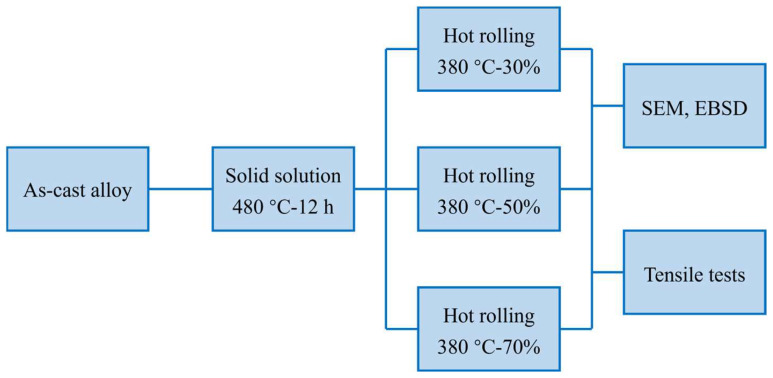
A schematic diagram of the processing route used in this work.

A CMT5105 electronic universal testing machine was used to perform room temperature tensile tests. The loading rate is 0.5 mm/s and tensile test samples are dog-bone shaped, having a gage length of 7 mm. To obtain reliable results, three specimens were tested for each state. Tensile loading was applied along the rolling direction (RD) for the hot rolled samples. The microstructures were characterized by a ZEISS G300 scanning electron microscopy (SEM, Zeiss, Oberkochen, Germany) equipped with an energy dispersive spectroscopy (EDS) and an electron backscatter diffractions system (EBSD). Channel 5 software was employed to post-process the EBSD data. All of the EBSD results shown in this paper were obtained through using this software. The samples for microstructure characterization were prepared according to the procedure given in ref [30]. The microstructure observations were conducted on the cross-section perpendicular to the normal direction (ND). A schematic diagram of the processing route used in this work is shown in Figure 1.

## 3. Results

### 3.1. Microstructures

**Figure 2:**
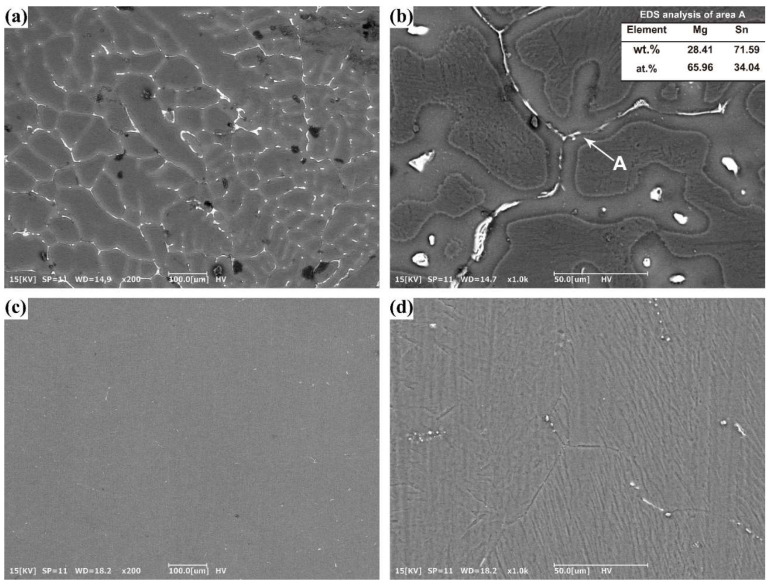
SEM images of (**a**,**b**) as-cast Mg-5Sn alloy; and (**c**,**d**) solid solution treated Mg-5Sn alloy. The inset in (**b**) is the result of EDS analysis of area A.

Figure 2 shows the SEM images of the as-cast and solid-solution treated Mg-5Sn alloys. Note that the as-cast Mg-5Sn alloy contains α-Mg matrix and intermetallic particles. According to previous studies [9,31,32,33], the intermetallic particles in the Mg-Sn binary alloys are Mg_2_Sn phase. The EDS analysis shown in Figure 2b confirms that the intermetallic compounds are Mg_2_Sn phase. α-Mg dendrites are very coarse with an arm spacing ranging from tens of microns to hundreds of microns. The Mg_2_Sn intermetallic compounds exhibit plate-like morphology. They are very coarse, and some of them have a length larger than 100 μm. The solid-solution treatment dissolved most of the Mg_2_Sn particles. Few of the Mg_2_Sn particles can still be observed near the grain boundaries, but their size is much smaller than that in the as-cast alloy, consistent with the previous studies [14,15].

**Figure 3:**
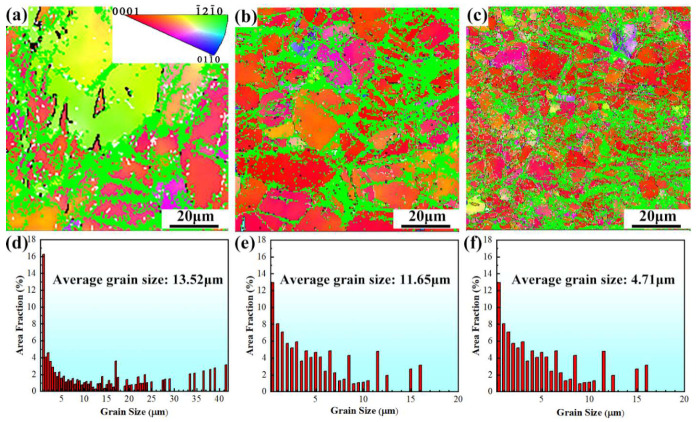
IPF maps of (**a**) R30 alloy; (**b**) R50 alloy; and (**c**) R70 alloy. Statics of grains in (**d**) R30 alloy; (**e**) R50 alloy; and (**f**) R70 alloy.

**Figure 4:**
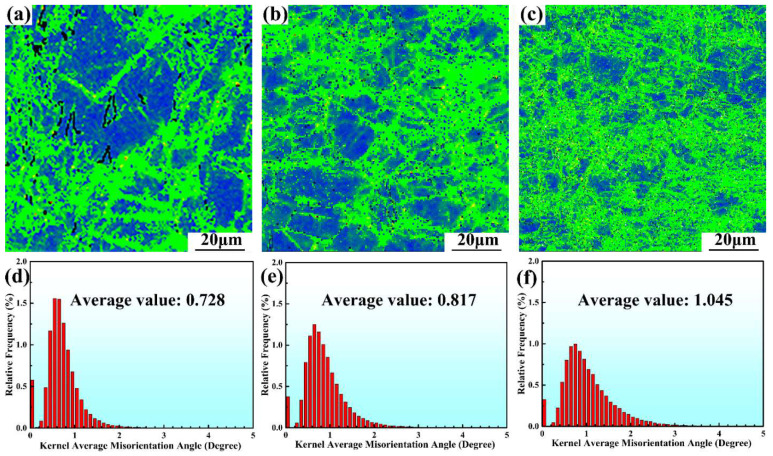
KAM maps of (**a**) R30 alloy; (**b**) R50 alloy; and (**c**) R70 alloy. Statics of KAM angle in (**d**) R30 alloy; (**e**) R50 alloy; and (**f**) R70 alloy.

Figure 3 shows the inverse pole figure (IPF) maps of the hot rolled alloys, along with the corresponding grain size distribution. The grains are colored by their crystallographic orientations with red, blue, and green denoting [0001], $\left[01\bar{1}0\right]$, and $\left[\bar{1}2\bar{1}0\right]$ directions, respectively. The hot rolled alloys possess much finer grains than the as-cast alloy. The grain size decreases from 13.52 μm to 11.65 μm and further to 4.71 μm, with the increase of the rolling reduction from 30% to 50% and further to 70%. The grain refinement is attributed to dynamic recrystallization (DRX) during hot rolling. As rolling reduction increases, the plastic deformation becomes severer, and the strain inside the alloy becomes larger. The strain distribution within the alloy can be estimated by kernel average misorientation (KAM) maps, because the severely deformed regions usually exhibit higher dislocation densities and thus larger KAM values [34,35]. The KAM images of the hot rolled alloys are shown in Figure 4. Noticeably, the average KAM value increases continuously with the increase of the rolling reduction from 30% to 70%, indicating the increase in dislocation density with the increase in rolling reduction. A higher dislocation density provides a larger driving force for DRX [36]. Increasing the rolling reduction can promote DRX and grain refinement [25]. Therefore, as the rolling reduction increases from 30% to 70%, the grain size decreases continuously.

**Figure 5:**
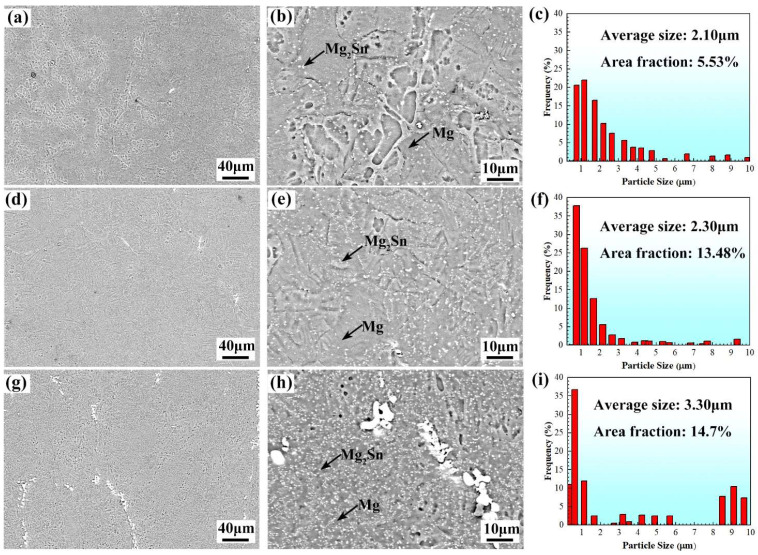
SEM images of (**a**,**b**) R30 alloy; (**d**,**e**) R50 alloy; and (**g**,**h**) R70 alloy. Statics of Mg_2_Sn phase in (**c**) R30 alloy; (**f**) R50 alloy; and (**i**) R70 alloy.

The SEM images of the hot rolled Mg-5Sn alloys are exhibited in Figure 5. Numerous fine particles can be observed in all three alloys. These fine particles are Mg_2_Sn precipitates formed during hot rolling through dynamic precipitation. The previous studies [15,37,38] demonstrate that plastic deformation can induce the dynamic precipitation of Mg_2_Sn phase. Our observation is consistent with previous studies [15,37,38]. Moreover, some coarse undissolved Mg_2_Sn particles exist in the alloys. The morphology of the undissolved Mg_2_Sn particles changes from plate-like in the as-cast alloy to “blocky” and their size is much smaller. Some of the undissolved Mg_2_Sn particles were fragmentized during hot rolling, as shown in Figure 5h.

It can be noted from Figure 5 that the size and area fraction of Mg_2_Sn particles are affected by rolling reduction. The average size and area fraction of Mg_2_Sn particles in the R30 alloy are 2.10 μm and 5.53%, respectively. As the rolling reduction increases to 50%, the average size of Mg_2_Sn particles increases slightly to 2.30 μm, but their area fraction rises to 13.48% (Figure 5f). With the further increase of the rolling reduction to 70%, the average size and area fraction of Mg_2_Sn particles increase to 3.30 μm and 14.7%, respectively. The formation of the Mg_2_Sn precipitates is due to dynamic precipitation during thermomechanical processing, as reported in previous studies [15,37,38]. The dislocations formed during plastic deformation can not only provide nucleation sites for the dynamic precipitation of the Mg_2_Sn phase, but also increase the diffusion rate of the Sn atoms [15,37]. When the rolling reduction is 30%, the dislocation density is relatively low (Figure 4d) and therefore the area fraction of Mg_2_Sn precipitates is relatively low. As the rolling reduction increases to 50%, more dislocations are accumulated in the alloy (Figure 4e), providing more nucleation sites for Mg_2_Sn phase. In addition, the pre-existing Mg_2_Sn precipitates may act as nucleation sites. Consequently, the area fraction of Mg_2_Sn particles for R50 alloy rises to 13.48% and their average size increases slightly. A further increase of the rolling reduction to 70% generates more dislocations (Figure 4f) and thus leads to the further increase in the average size and area fraction of Mg_2_Sn particles.

### 3.2. Mechanical Properties

**Figure 6:**
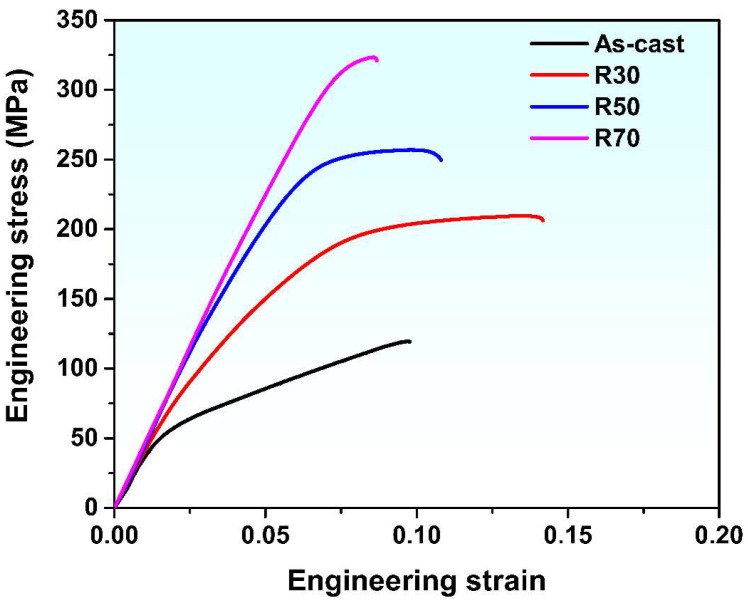
Engineering stress–engineering strain curves of as-cast and hot rolled alloys.

**Figure 7:**
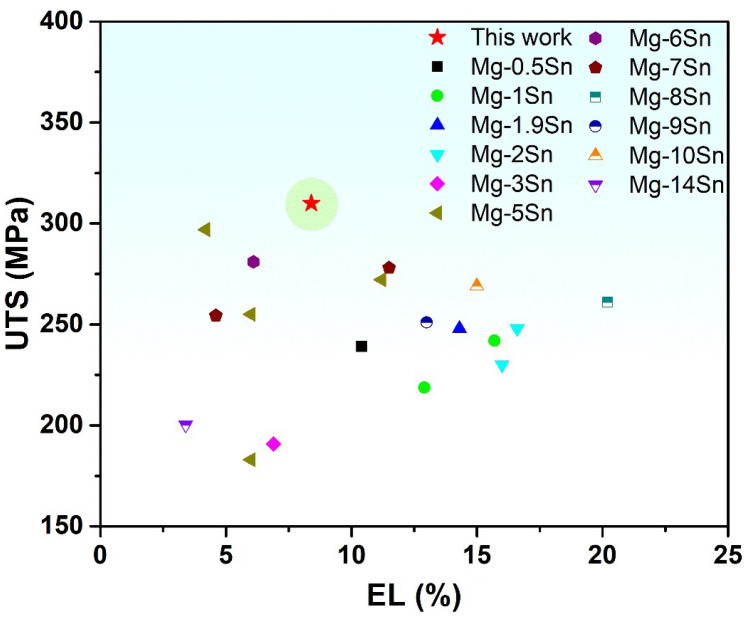
Mechanical performance comparison between the R70 alloy developed in this work and recently developed hot rolled Mg-Sn alloys [14,15,17,18,19,42,43,44,45,46,47,48].

Engineering the stress–strain curves of the as-cast and hot rolled alloys are presented in Figure 6. Their YS, UTS, and EL are presented in Table 1. It can be noted that the strengths of hot rolled alloys are much higher than that of the as-cast alloy. In addition, YS and UTS of the hot rolled alloys increase continuously with increasing rolling reduction, whereas the EL of the alloys shows an opposite trend. The R70 alloy has the highest UTS of 310 MPa, but the lowest EL of 8.4%. The R30 alloy possesses a much better ductility than the as-cast alloy while the R50 and R70 alloys have a ductility similar to that of the as-cast alloy. The mechanical performance of the R70 alloy is compared with that of the recently developed hot rolled Mg-Sn alloys, as shown in Figure 7. It can be noted that the R70 alloy exhibits good comprehensive mechanical properties.

The enhanced strength of the hot rolled alloys can be ascribed to precipitation strengthening, grain refinement strengthening, and dislocation strengthening. The strengthening effect of fine and dense precipitates is stronger than that of coarse and sparse particles [39]. The finer grains can exert a stronger strengthening effect than coarse grains [40]. Dislocations of a higher density results in a stronger dislocation strengthening [41]. The coarse grains and Mg_2_Sn phase in the as-cast alloy (Figure 2) are less effective in strengthening the alloy compared to the relatively fine grains and Mg_2_Sn precipitates in the hot rolled alloys (Figure 3 and Figure 5). In addition, the plastic deformation induces the accumulation of dislocations in the hot rolled alloys, as indicated by the KAM maps shown in Figure 4. Therefore, the hot rolled alloys have a much higher strength than the as-cast alloy. As the rolling reduction increases from 30% to 70%, the grain size decreases while the area fraction of Mg_2_Sn particles and dislocation density (as implied by the KAM value) increase continuously. Therefore, the strengths of the hot rolled alloys exhibit a rising trend with increasing rolling reduction.

According to the Hall–Petch relation [49], the yield strength $\sigma_{HP}$ is related to grain size *d* by:

(1) $$\sigma_{HP}=\sigma_{0}+kd^{-0.5}$$

where $\sigma_{0}$ is lattice friction stress; $\sigma_{0}=11\;\mathrm{MPa}$ [50]; and *k* is the Hall–Petch slope, $k=280\;\mathrm{MPa}\sqrt{\mu m}$ [8]. Substituting the values of the grain size into Equation (1), we can obtain the $\sigma_{HP}$ for as-cast and hot rolled alloys. Subtracting the $\sigma_{HP}$ for the as-cast alloy from that for the hot rolled alloys, we can obtain the strength enhancement induced by grain refinement strengthening $\Delta \sigma_{HP}$. $\Delta \sigma_{HP}$ for the R30, R50, and R70 alloys were calculated to be 48.1, 54.2, and 100.9 MPa, respectively.

The strength increment caused by dislocation strengthening $\Delta \sigma_{d}$ can be estimated by the Bailey–Hirsch equation [51]:

(2) $$\Delta \sigma_{d}=M\alpha Gb\sqrt{\rho }$$

where *M* is the Taylor factor (*M* = 2.1 [52]); α is the Taylor constant (α = 0.2 [50]); *G* is the shear modulus of Mg (*G* = 16.6 GPa [53]); *b* is the Burgers vector for basal slip of Mg (*b* = 0.321 nm [53]); and ρ denotes the dislocation density of the alloy. ρ can be evaluated by [54]:

(3) $$\rho =\frac{2\theta }{ub}$$

where θ represents the local misorientation angle obtained from the EBSD KAM maps (Figure 4) and *u* is the EBSD step size. *u* is 0.6 μm for the R30 and R50 alloys and 0.3 μm for R70 alloy. Combining Equations (2) and (3), the $\Delta \sigma_{d}$ for the R30, R50, and R70 alloys can be obtained as 25.7, 27.2, and 43.6 MPa, respectively.

Strength enhancement arising from precipitation strengthening $\Delta \sigma_{p}$ can be calculated by the Orowan–Ashby equation [55]:

(4) $$\Delta \sigma_{p}=\frac{0.13Gb}{\lambda }ln\frac{d_{p}}{2b}$$

where λ is the interparticle distance and $d_{p}$ is the average particle size. $\Delta \sigma_{p}$ for the R30, R50, and R70 alloys were estimated to be 5.5, 9.7, and 18.6 MPa, respectively.

The total strength increment Δσ induced by the three strengthening mechanisms can be calculated by:

(5) $$\Delta \sigma =\Delta \sigma_{HP}+\Delta \sigma_{d}+\Delta \sigma_{p}$$

Δσ for the R30, R50, and R70 alloys were estimated to be 79.3, 90.9, and 163.2 MPa, respectively. As can be seen from Table 1, the YS of the R30, R50, and R70 alloys exceed that of the as-cast alloy by 74, 92, and 160 MPa, respectively. The calculated strength enhancement is in good agreement with experimental results. It can be noted from comparing values of $\Delta \sigma_{HP}$, $\Delta \sigma_{d}$, and $\Delta \sigma_{p}$ that grain refinement strengthening is the dominant strengthening mechanism, followed by dislocation strengthening.

**Figure 8:**
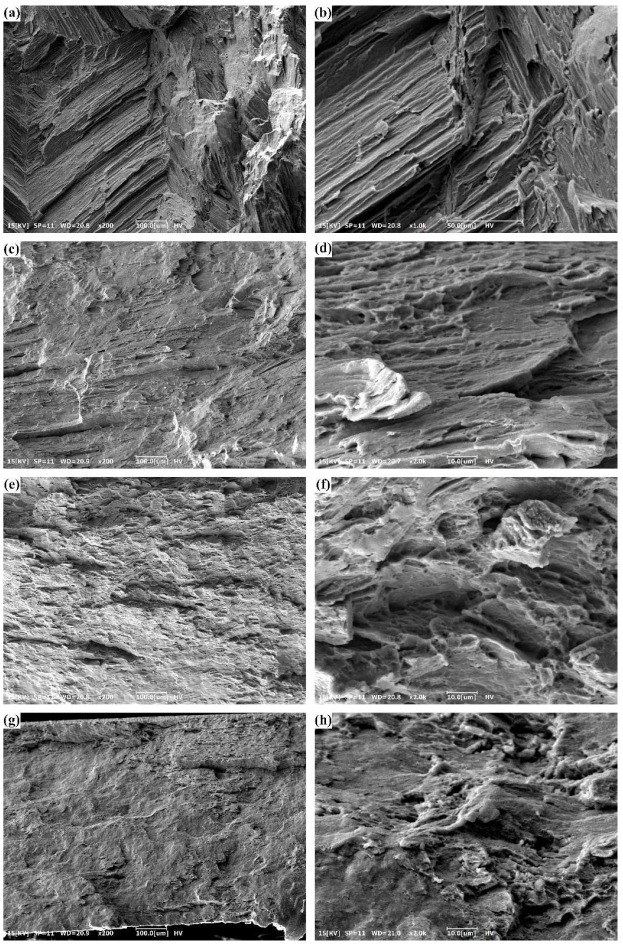
Fracture surfaces of (**a**,**b**) as-cast alloy; (**c**,**d**) R30 alloy; (**e**,**f**) R50 alloy; and (**g**,**h**) R70 alloy.

The fracture surfaces of the as-cast and hot rolled alloys are displayed in Figure 8. As-cast alloy exhibits the typical characteristics of brittle fracture, with many cleavage steps on the fracture surfaces. Dimples can be observed on the fracture surfaces of the R30 and R50 alloys, exhibiting to some extent the feature of a micro-void coalescence fracture. Therefore, the ductility of the R30 and R50 alloys are better than the as-cast alloy. As the rolling reduction increases to 70%, the cleavage steps and river patterns appear again, indicating the drop in ductility. The trend of ductility with the variation of rolling reduction deduced from fracture surfaces is consistent with that observed from the engineering stress–strain curves shown in Figure 6. The Mg_2_Sn phase is hard and brittle while the Mg matrix is relatively soft. Due to the deformation incompatibility, the stress concentration is induced at the interface between the coarse Mg_2_Sn particles and Mg matrix. When the stress concentration reaches a critical level, the interfacial cracks nucleate and propagate, leading the fracture of Mg-5Sn alloy [15,44,56].

The ductility of the metallic materials is also related to precipitates, grain size, and dislocation density. The grain boundaries, precipitates, and high-density dislocations can act as dislocation barriers and block dislocation movements, leading to dislocation accumulation and promoting stress concentration [57,58,59,60]. Therefore, fine grains, high-density precipitates, and high-density dislocations may generally have a negative influence on ductility. With the increase of rolling reduction from 30% to 70%, the area fraction of the Mg_2_Sn particles and dislocation density increase, and meanwhile the grain size decreases. Therefore, the EL of hot rolled Mg-Sn alloys shows a decreasing trend with increasing rolling reduction. As-cast alloy has poorer ductility compared to the R30 alloy, due to the presence of the coarse Mg_2_Sn phase which may act as crack nucleation sites.

## 4. Conclusions

The microstructure evolution and mechanical properties of a Mg-5Sn alloy which was hot rolled at three different rolling reductions (30%, 50%, and 70%) were investigated in this work. The grain size of hot rolled alloys decreases from 13.5 to 4.7 μm as rolling reduction increases from 30% to 70%. In contrast, the area fraction of the Mg_2_Sn particles and dislocation density rise with the increase in rolling reduction. Consequently, the strengths of the alloys exhibit an increasing trend with increasing rolling reduction, whereas the EL of the alloys shows a decreasing trend. Specifically, the UTS of the hot rolled alloys increases from 213 to 310 MPa while the EL of the hot rolled alloys decreases from 14.1% to 8.4% with the increase of rolling reduction from 30% to 70%. Strength enhancement is mainly ascribed to precipitation strengthening, grain refinement strengthening, and dislocation strengthening. Hot rolled alloys possess much better comprehensive mechanical properties than the as-cast alloy.

## Figures and Tables

**Table 1:** YS, UTS, and EL of as-cast and hot rolled Mg-5Sn alloys.

Alloys	YS (MPa)	UTS (MPa)	EL (%)
As-cast alloy	31	133	9.8
R30 alloy	105	213	14.1
R50 alloy	123	252	10.5
R70 alloy	191	310	8.4
